# Analyses of single-cell and bulk RNA sequencing combined with machine learning reveal the expression patterns of disrupted mitophagy in schizophrenia

**DOI:** 10.3389/fpsyt.2024.1429437

**Published:** 2024-09-17

**Authors:** Wei Yang, Kun Lian, Jing Ye, Yuqi Cheng, Xiufeng Xu

**Affiliations:** ^1^ Department of Psychiatry, The First Affiliated Hospital of Kunming Medical University, Kunming, Yunnan, China; ^2^ Department of Psychiatry, The Second People’s Hospital of Yuxi, Yuxi, Yunnan, China; ^3^ Yuxi Hospital affiliated to Kunming University of Science and Technology, Yuxi, Yunnan, China; ^4^ Department of Neurosurgery, The Second Affiliated Hospital of Kunming Medical University, Kunming, Yunnan, China; ^5^ Schizophrenia Research Program, Yunnan Clinical Research Center for Mental Disorders, Kunming, Yunnan, China

**Keywords:** schizophrenia, mitophagy, bulk RNA analysis, single-cell RNA analysis, machine learning

## Abstract

**Background:**

Mitochondrial dysfunction is an important factor in the pathogenesis of schizophrenia. However, the relationship between mitophagy and schizophrenia remains to be elucidated.

**Methods:**

Single-cell RNA sequencing datasets of peripheral blood and brain organoids from SCZ patients and healthy controls were retrieved. Mitophagy-related genes that were differentially expressed between the two groups were screened. The diagnostic model based on key mitophagy genes was constructed using two machine learning methods, and the relationship between mitophagy and immune cells was analyzed. Single-cell RNA sequencing data of brain organoids was used to calculate the mitophagy score (Mitoscore).

**Results:**

We found 7 key mitophagy genes to construct a diagnostic model. The mitophagy genes were related to the infiltration of neutrophils, activated dendritic cells, resting NK cells, regulatory T cells, resting memory T cells, and CD8 T cells. In addition, we identified 12 cell clusters based on the Mitoscore, and the most abundant neurons were further divided into three subgroups. Results at the single-cell level showed that Mitohigh_Neuron established a novel interaction with endothelial cells via SPP1 signaling pathway, suggesting their distinct roles in SCZ pathogenesis.

**Conclusion:**

We identified a mitophagy signature for schizophrenia that provides new insights into disease pathogenesis and new possibilities for its diagnosis and treatment.

## 1 Introduction

Schizophrenia is a multifaceted psychiatric disorder involving genetic, neurological, immunological and other factors, although its exact pathogenesis is still unclear (1). Most of the genetic factors related to schizophrenia that have been identified so far are common alleles with small effects, although there are reports of rare copy number and coding variants (2–4). The involvement of these specific genes and loci point to complex biological mechanisms.

Recent studies have shown that schizophrenia may be associated with mitochondrial dysfunction, particularly aberrant mitophagy (5, 6). Mitochondria are the main energy source of cells and play an important role in physiological processes such as apoptosis and oxidative stress (7, 8). Mitochondrial dysfunction is closely related to the occurrence and development of multiple neurological diseases, especially mental disorders (5, 9–12). In fact, mitochondrial DNA damage and reduced mitochondrial membrane potential (MMP) have been observed in the brain tissues of schizophrenia patients (13–15). These abnormalities can disrupt mitophagy and increase apoptosis, thereby affecting neurological functions.

The aim of this study was to elucidate the role of aberrant mitophagy genes in the pathogenesis of schizophrenia. To this end, we analyzed the transcriptomic profiles of healthy controls and schizophrenia patients and screened the mitophagy-related genes potentially involved in the pathogenesis of schizophrenia using multiple machine learning approaches. The mitophagy signature identified in this study can be a potential diagnostic marker or therapeutic target for schizophrenia.

## 2 Materials and methods

### 2.1 Study design

**Figure 1:**
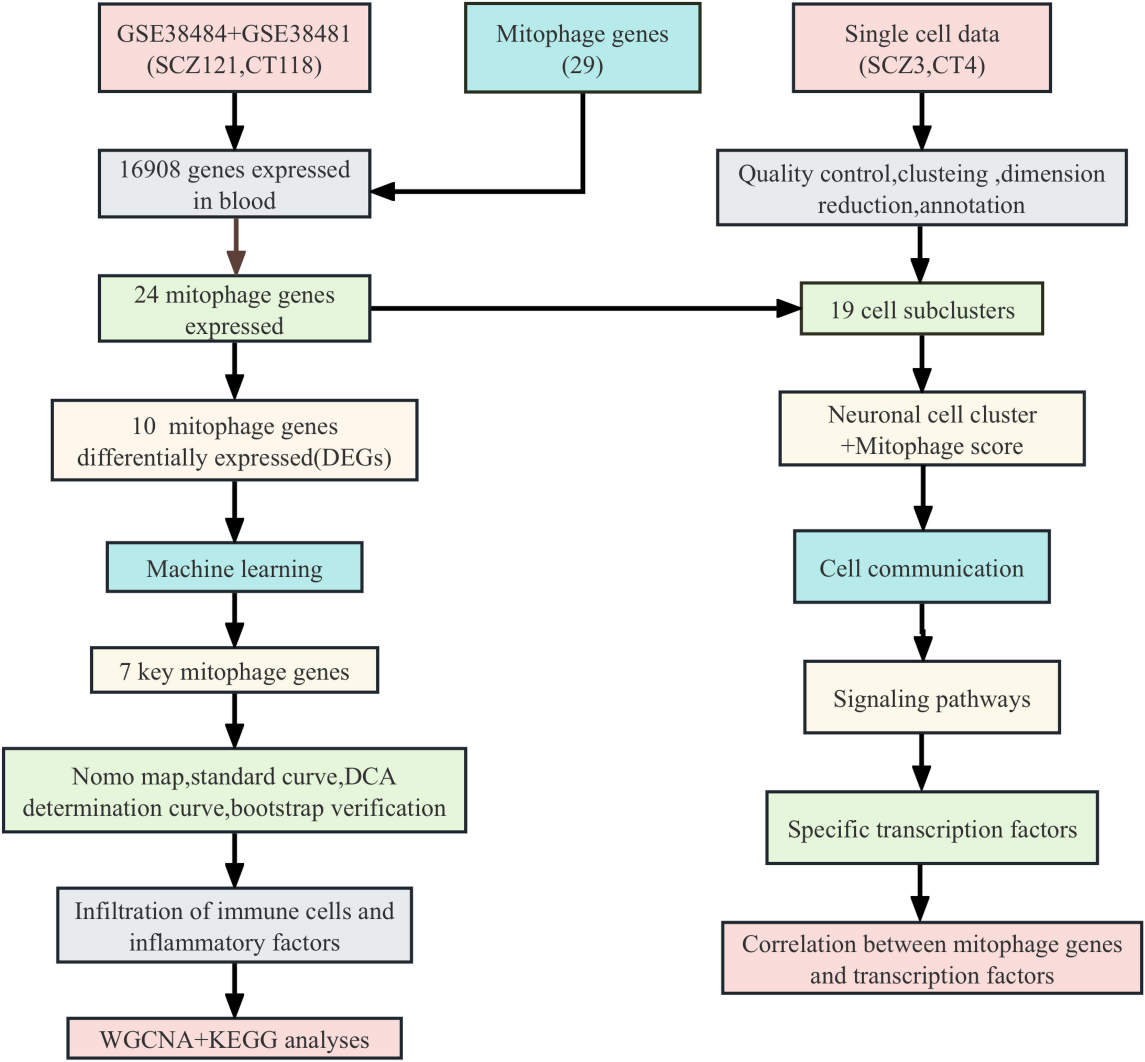
Flow chart of this study.

The role of mitophagy genes in the pathogenesis of schizophrenia were explored using machine learning methods such as support vector machine (SVM) and random forest (RF), cluster analysis such as weighted correlation network analysis (WGCNA) and fast gene set enrichment analysis (fsGSEA), and single-cell transcriptomic analysis. The study design is shown in **Figure 1**.

### 2.2 Data retrieval

The schizophrenia datasets GSE38484 (GPL6947 Illumina HumanHT-12 V3.0 microarray) and GSE38481 (GPL6883 Illumina HumanRef-8 V3.0 microarray) were retrieved from the NCBI Gene Expression Omnibus (GEO) database. The mitophagy gene dataset was downloaded from an online source (https://www.gsea-msigdb.org/) (16) (**Supplementary Table 1**). The GSE184878 dataset including the single-cell RNA-sequencing (scRNA-seq) data of four normal and 4 3D brain organoid SCZ samples was also downloaded.

### 2.3 Differential expression analysis

The GSE38484 and GSE38481 datasets were corrected, normalized, and merged using the “limma” package in R to obtain a new dataset of 121 schizophrenia patients and 118 healthy controls (17). Genes with a p-value < 0.05 and |Log2FC (fold-change)| > 1.5 were considered differentially expressed genes (DEGs) for further analysis. Heatmaps of the DEGs was generated using the “heatmap” and “ggplot2” R packages, respectively. We identified Mito-DEGsby finding common genes between DEGs and mitophage genes.

### 2.4 Gene set enrichment analysis

We employed the Molecular Signatures Database (MSigDB) to obtain gene sets related to Homo sapiens in the “Hallmark” category using the “msigdbr” R package. Gene set variation analysis (GSVA) was performed using the “GSVA” package with the single-sample gene set enrichment analysis (ssGSEA) approach using a Gaussian kernel cumulative distribution function. GSEA was used to compare the mitophagy pathways between SCZ and control samples based on the Kyoto Encyclopedia of Genes and Genomes (KEGG) database.

### 2.5 Identification of key genes through machine learning

Multiple machine-learning techniques were used to make predictions about disease states. Support vector machine (SVM) is a supervised learning model and related learning algorithm for analyzing data in classification and regression analysis. Random forest is a widely used supervised learning model in ensemble learning. This algorithm integrates the outputs of multiple decision trees to generate a single result. SVM and RF machine learning techniques were used to screen the potential mitophagy genes with diagnostic potential, and the top ranked genes in each gene were intercrossed to obtain the most potential candidate genes. Subsequently, bootstrap method was used to establish a multiple regression diagnostic model for internal validation.

### 2.6 Relationship between key genes and immune infiltration

To explore the immune landscape of schizophrenia based on mitophagy gene expression profiles, we employed the CIBERSORT algorithm (18). The algorithm is based on gene expression profiling to estimate the type and abundance of immune cells in a mixed cell population. This estimate result provides an understanding of the immune cells in SCZ samples. Further to evaluate and visualize the differences and correlations of immune cell infiltration between SCZ and control samples, we used the “corplot” and “vioplot” functions in the IOBR software package to evaluate the correlation between immune cell types and seven key genes (19). In addition, the association between inflammatory factors and diagnostic genes was analyzed. The results were visualized using the “ggplot2” package in R (20).

### 2.7 Calculation of Mitoscore

To better evaluate the correlation of mitophagy genes among different cell subsets, we calculated the signal-to-noise ratio changes of different mitophagy genes at the single-cell level (21). Briefly, the average normalized TPM values were calculated for each gene from the scRNAseq data, and the genes were divided into 50 expression bins using a random sampling method with 1000 replicates. Randomly characterized genes were selected from each bin. According to the relevant formula, Mitoscores belonging to normal distribution or mixed normal distribution were determined. Mitoscore is the fractional level used to quantify the expression of mitophagy genes in individual cells or samples.

The Mitoscore was calculated by analyzing the expression levels of specific gene sets associated with the mitophagy process. We first identified a list of genes associated with mitophagy and then calculated the expression levels of these genes in each cell. By averaging or weighting the expression levels of these genes, we obtained a Mitoscore for each cell. This score reflects the overall expression level of mitophagy genes and can be used to compare mitophagy activity between different cells, samples, or populations. In our study, we used Mitoscore to identify and distinguish subsets of cells with different mitophagy activities.

### 2.8 Classification of mitophagy subtypes

Consensus clustering is mainly used to determine the possible large number of members in the data set and find new disease molecular subclasses. The 24 mitophagy genes were clustered using the R package ConsensusClusterPlus, and scored using calscore (22). Two stable gene subtypes were obtained, and the heat map was drawn. The differences in age, gender, predictive value, expression levels of mitophagy genes, immune cells and inflammatory factors were also analyzed. The plots were drawn using ggstatsplot and ggboxplot.

### 2.9 Multiple reclustering analysis

The abnormal genes and samples were removed using the goodSamplesGenes method of the R package WGCNA (23). The scale-free co-expression network was constructed using WGCNA, and the sensitivity was set to 3. The similarities and differences of module characteristic genes were calculated, a tangent line for the module dendrogram was selected, and some modules were merged. In addition, modules with a distance of less than 0.5 were incorporated, resulting in 12 co-expressed modules. The gene sets that could not be assigned to any module formed the grey module. The two mitophagy subtypes were subjected to fGSEA, and the differentially enriched pathways were analyzed using https://metascape.org.

### 2.10 Single cell analysis

The scRNA-seq data of 3D brain organoids from schizophrenia patients and healthy controls were downloaded from the GSE184878 dataset. The SeuratR v4.4.0 was used for for single-cell analysis such as quality control, dimensionality reduction, clustering, and more. The cells with less than 200 genes or more than 2500 genes were excluded to obtain genes with high-fold changes in expression. The predominant cell types were identified using the automated cell type annotation tool SingleR v1.4.116 (24). The transcriptomic profile of each cell was compared with the built-in reference data set in celldex v1.0.0 index 16, and selected the cell type markers using the MonacoImmuneData function. The cell types were extracted from the pooled dataset and subjected to PCA, UMAP, and SNN analyses using the same parameters as previously. The FindAllMarkers function in Seurat was applied to each selected cluster for each selected principal cell type to analyze the expression of the cluster-related genes. Inflammatory genes were obtained from the Molecular Signature database v7.5 HALLMARK_INFLAMMATORY_RESPONSE (25). The mitophagy gene scores of the individual cells in schizophrenia and control samples were obtained by pseudo-time trace analysis using the R package Monocle 3 (26). Finally, the neurons were classified into the Mitohigh_Neuron, Mitomedian_Neuron, and Mitolow_Neuron subgroups based on high, medium, and low mitophagy gene scores respectively.

### 2.11 Analysis of intercellular communication

Cell-cell interaction was analyzed in terms of the expression of ligands/receptors between different cell types using the CellphoneDB software package (27). The number of statistical iterations in the scRNA-seq count matrix was set to 1000, and genes expressed in less than 10% of the cells in each cluster were eliminated. The CellPhoneDB repository was used to identify the cell-cell interactions, and P value < 0.05 was the threshold for significance. Average receptor expression levels in clusters and average ligand expression levels in interacting clusters were calculated. Network plots were used to illustrate the differences between the ligand-receptor interactions of the Mitohigh_Neuron, Mitomedian_Neuron, and Mitolow_Neuron groups.

### 2.12 SCENIC analysis of Mitoscore groups

Since the gene regulatory network(GRNs) dominated by transcription factors (TFs) plays an important role in the transcriptional state of cells, we used the Python-based pySCENIC package (version: 0.12.0)to analyze the gene regulatory network of TFs (28).Single-cell regulatory network inference and clustering (SCENIC) is a GRNs algorithm developed specifically for single-cell data. Its innovation lies in the introduction of gene co-expression networks inferred by TF motif sequence validation statistical methods, thereby identifying highly reliable GRNs dominated by TFs.The algorithm was first used to identify modules of co-expressed genes and TFs from the count matrix of scRNA-seq data. These modules were then trimmed by cis-regulatory motif identification of possible target genes using RcisTarget. Finally, the activity of each TF and potential target gene in each cell was quantified and plotted using recovery analysis. The difference in TF activity scores between the Mitohigh_Neuro and Mitolow_Neuro groups was visualized with a heatmap.

### 2.13 Statistical analysis

All analyses were performed using R version 4.2.1. The relative abundance of major cell types and clusters were expressed as percentages using the R package ggpub v0.4.0. Wilcoxon rank sum test was used to compare the measurement data. Spearman correlation analysis was used to determine the correlation coefficient between variables. Receptor operating characteristic (ROC) curves were plotted and the area under the curve (AUC) values were calculated to evaluate the diagnostic performance of variables. *P* < 0.05 was considered statistically significant.

## 3 Results

### 3.1 Differential expression analysis of mitophagy genes

**Figure 2:**
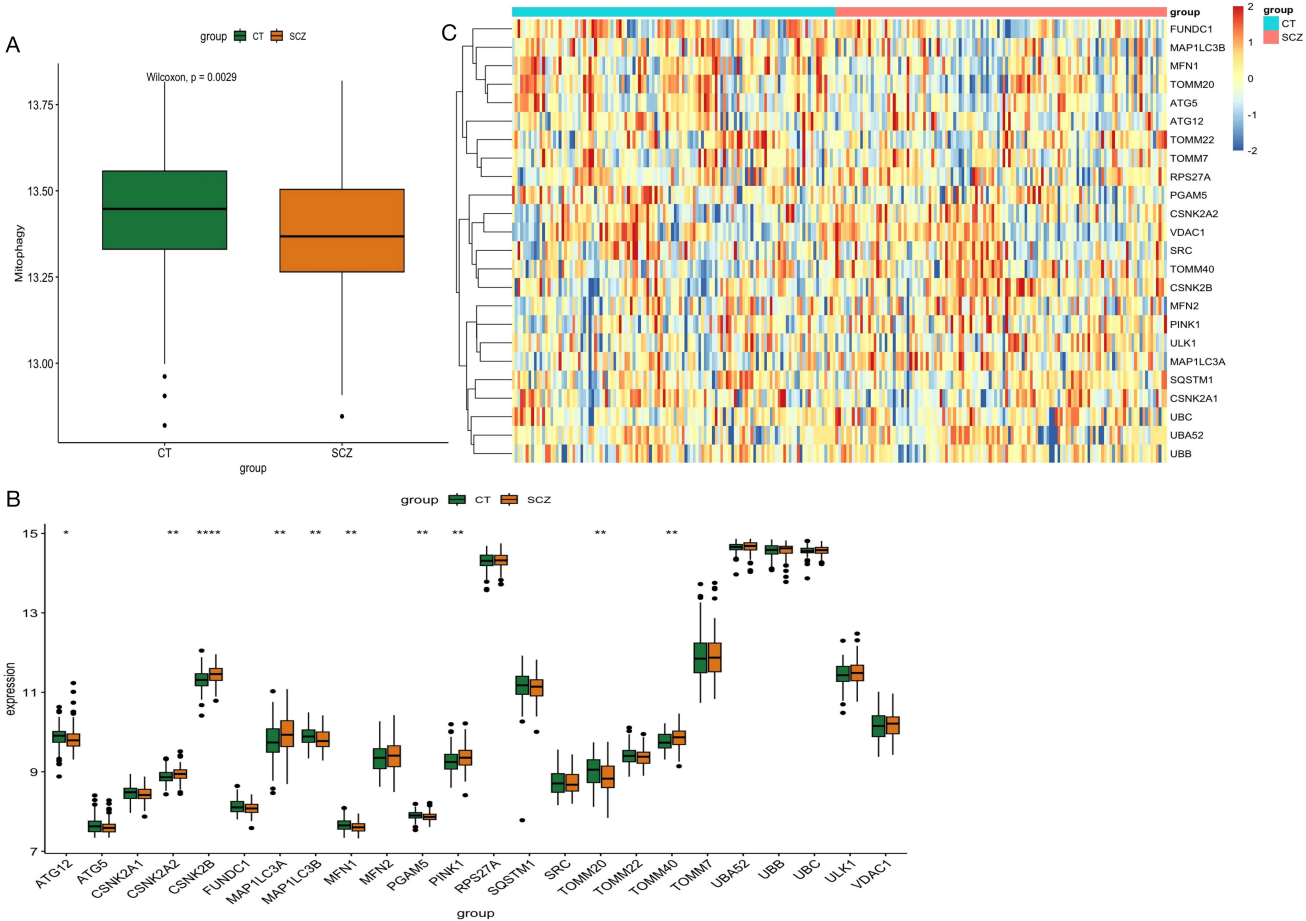
Differential expression analysis of GSE38484 and GSE38481 after merging. **(A)** GSVA analysis of mitophage-related gene between SCZ and control. **(B)** Bar graph of 24 mitophagy genes between SCZ and CT groups. **(C)** Heat map of 24 mitophage genes between SCZ and CT groups. (*p < 0.05; **p < 0.01; ****p < 0.0001).

**Figure 3:**
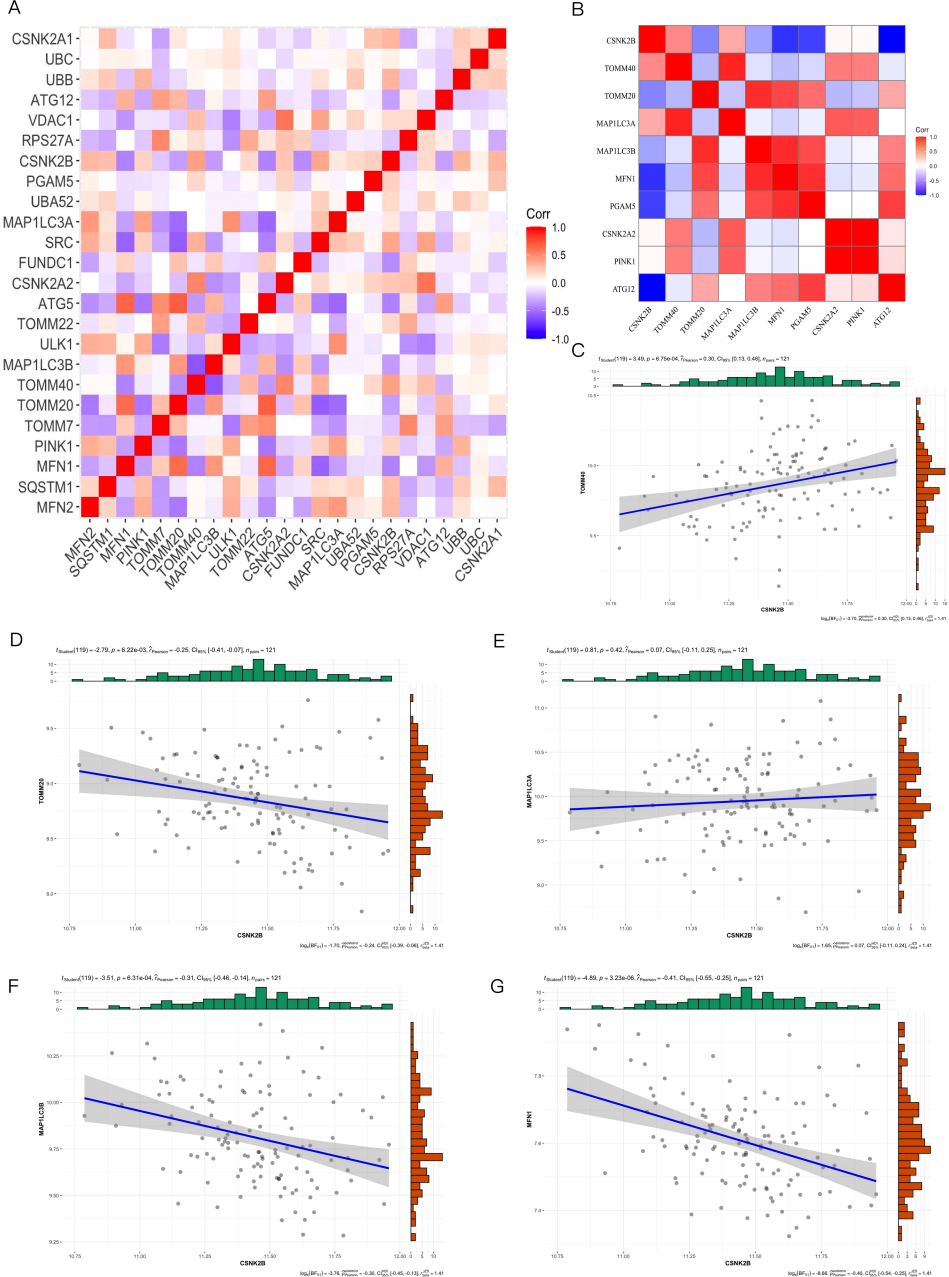
Mitophagy Gene Interrelationships. **(A)** Heat map of 24 mitophagy genes expressed in blood; **(B)** Heat map of the 10 mitophagy genes differentially expressed; **(C)** Correlation between CSNK2B and TOOM40; **(D)** Correlation between CSNK2B and TOOM20; **(E)** Correlation between CSNK2B and MAP1LC3A; **(F)** Correlation between CSNK2B and MAP1LC3B; **(G)** Correlation plot of CSNK2B and MFN1.

After removing batch effects from the combined data set of 239 samples, we generated a comprehensive gene expression profile of 16908 genes, of which 24 mitochondrial genes were expressed in this gene expression profile (**Supplementary Table 2**). GSVA analysis revealed a significant increase in mitophagy scores in SCZ patients compared with controls (**Figure 2A**). We identified 2,844 differentially expressed genes, and only 10 mitophagy genes were differentially expressed between the SCZ and control groups (**Figure 2B**) (**Supplementary Table 3**). For example, CSNK2B expression was upregulated in SCZ, whereas TOMM20 expression was downregulated (**Figure 2C**). Furthermore, the correlation coefficient between the 24 mitophagy genes and 10 mitophage DEGs were also calculated (**Figures 3A, B**). CSNK2B showed the most significant differential expression, and was positively correlated with TOMM40 (p=6.75e-04), and negatively with TOMM20 (p=6.22e-03), MAP1LC3B (p=6.31e-04) and MFN1 (p=3.23e-06) (**Figures 3C–G**).

### 3.2 Construction of diagnostic mitophagy signature

**Figure 4:**
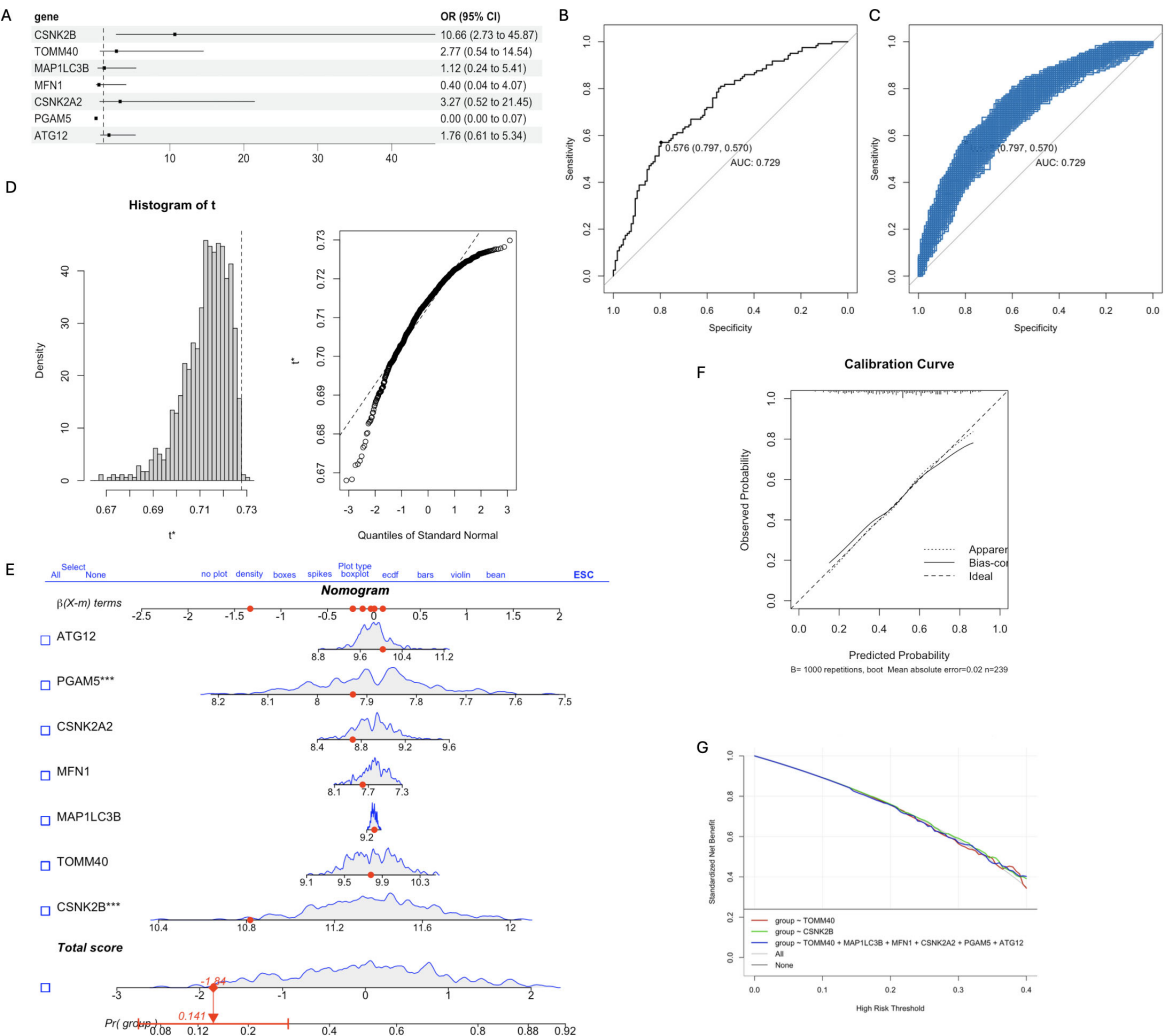
The diagnostic model was constructed based on the 7 key mitophagy genes obtained by machine learning. **(A)** Multivariate logistic regression OR value and 95%CI nomogram; **(B)** ROC curve of diagnostic model, AUC=0.729; **(C)** The Bootstrap validation results of ROC performance was repeated for 1000 times; **(D)** Histogram of AUC principal component distribution (left panel) and standard normal value quantile histogram (right panel); **(E)** Nomogram was used for model validation. **(F)** Calibration curves were used to evaluate the predictive accuracy of the nomogram; **(G)** Decision curve analysis demonstrating clinical benefit of the nomogram. (*p < 0.05; ***p < 0.001).

As shown in **Figure 4A**, the key mitophagy genes identified by both SVM and RF were CSNK2B (OR=10.66, 95% CI=2.73-45.8), TOMM40 (OR=2.77, 95% CI=0.54-14.54), MAP1LC3B (OR=1.12, 95% CI=0.24-5.41), MFN1 (OR=0.4, 95% CI=0.04-4.07), CSNK2A2 (OR=3.27, 95% CI=0.52-21.45), PGAM5 (OR=0.00, 95% CI=0.00-0.07) and ATG12 (OR=1.76, 95% CI=0.61-5.34). The AUC of the diagnostic model constructed using 7 key mitophage genes was 0.729, indicating good diagnostic efficiency (**Figure 4B**). Furthermore, we performed an internal validation with 1000 replicates using Bootstrap, and its performance is shown in **Figure 4C**. The histogram of AUC and quantiles of standard normal are shown in **Figure 4D**. In addition, we used Nomogram for model validation (**Figure 4E**), Pr(group)=0.141, indicating a high probability of diagnosis as a CT sample. The calibration curve evaluated the predictive accuracy of the nomogram (**Figure 4F**). The decision curve showed that the diagnostic efficacy of TOMM40 and CSNK2B alone was similar to that of the 7 key genes model (**Figure 4G**), indicating their potential as independent diagnostic markers.

### 3.3 Analysis of immune landscape associated with mitophagy

**Figure 5:**
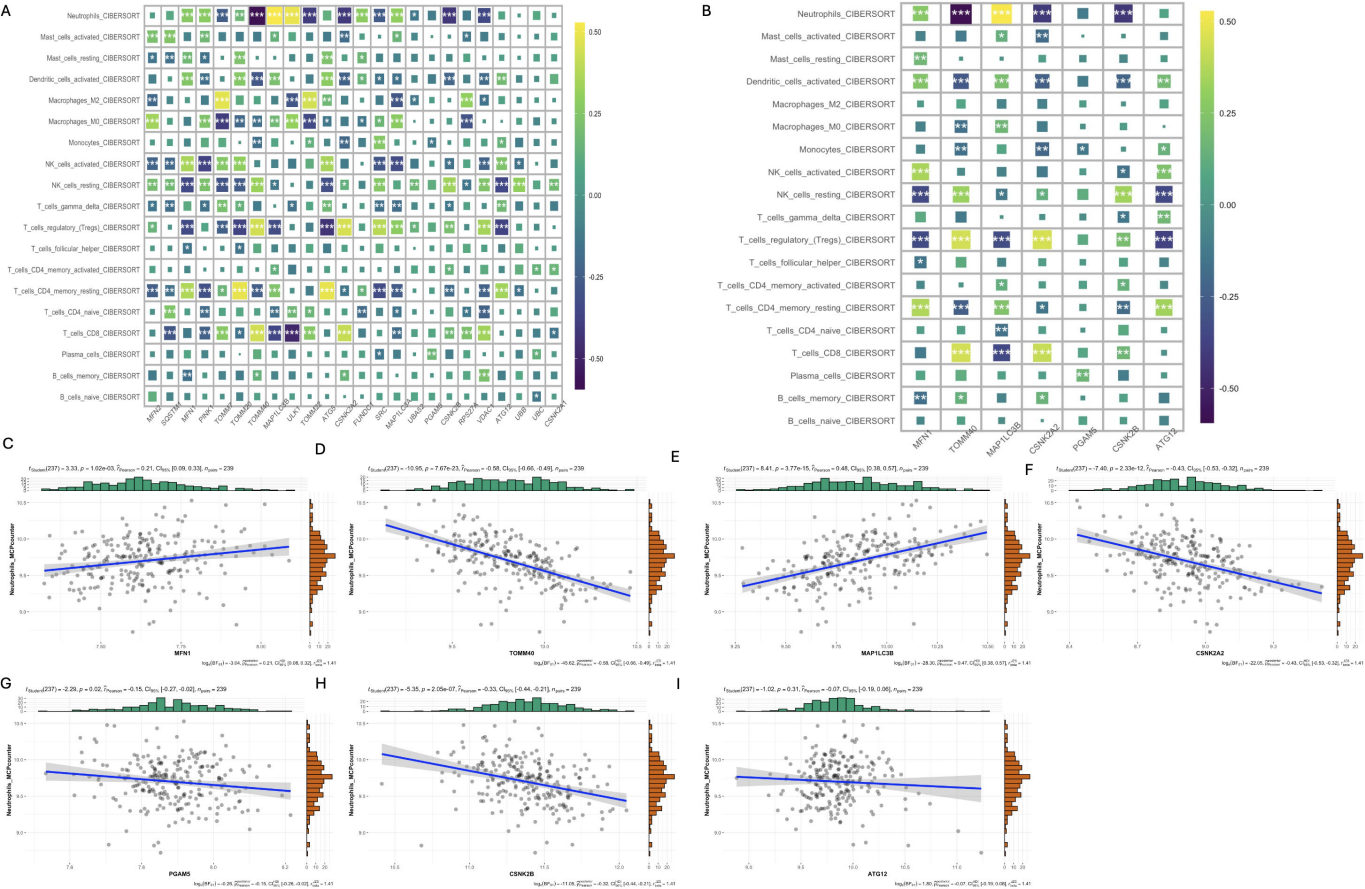
Analysis of the relationship between mitophagy genes and immune cell infiltration. **(A)** Heat map of CIBERSORT correlation analysis between 24 mitophagy genes and 19 immune cells; **(B)** Heat map of CIBERSORT correlation between 7 key mitophagy genes and 19 immune cells; **(C-I)** Correlation analysis of seven top mitophagy genes: MFN1, TOMM40, MAP1LC3B, CSNK2A2, PGAM5, CSNK2B, ATG12 and Neutrophils_MCPcounter abundance. (*p < 0.05; **p < 0.01; ***p < 0.001).

**Figure 6:**
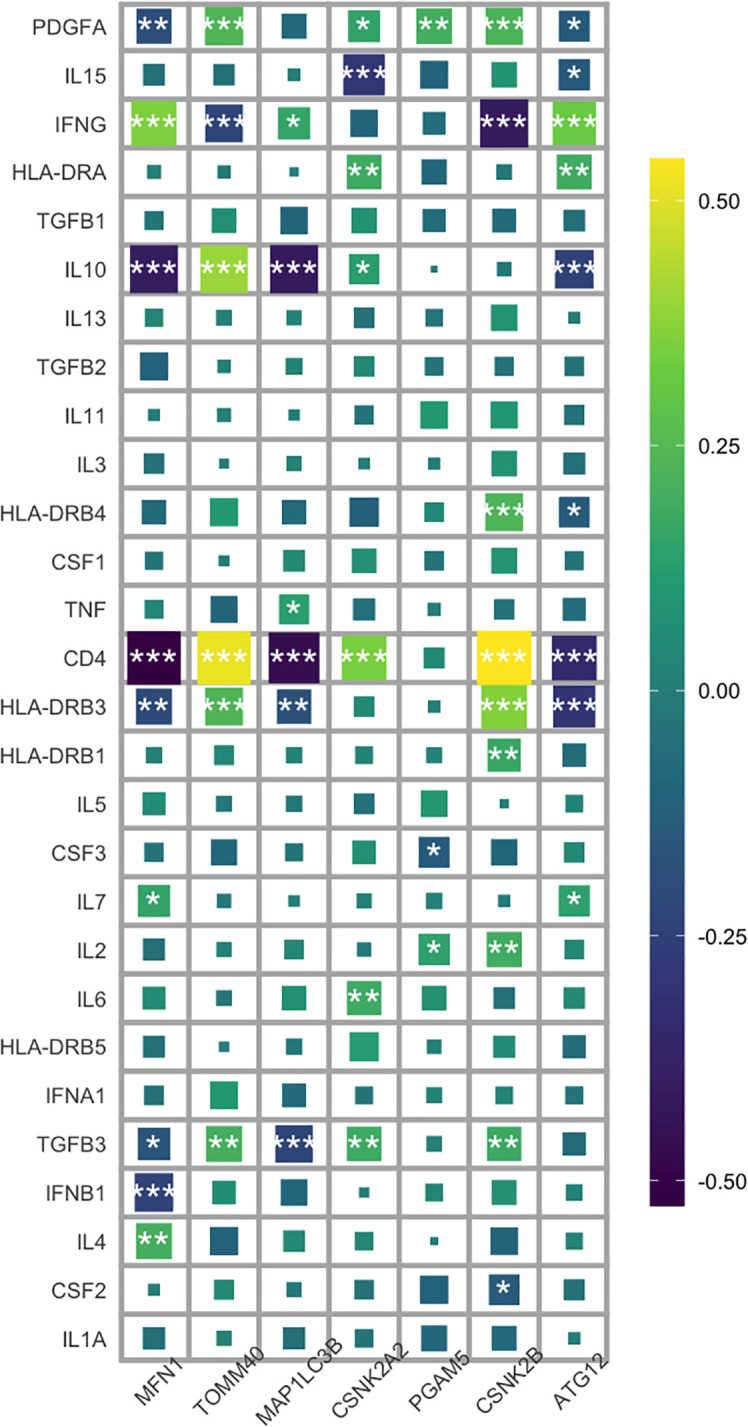
Heat map analysis of correlation between 7 key mitophagy genes and 28 inflammatory factors. (*p < 0.05; **p < 0.01; ***p < 0.001).

Neuroimmune responses play a key role in the occurrence and development of schizophrenia. Therefore, we evaluated the relationship between mitophagy genes and the relative distribution of 22 immune cells using the CIBERSORT algorithm. As shown in **Figure 5A**, the mitophagy genes were significantly associated with neutrophils, activated dendritic cells (DCs), activated NK cells, resting NK cells, regulatory T cells, activated CD4 memory T cells, and CD8 T cells. MFN1 (r=0.21, p=1.02e-03) and MAP1LC3B (r=0.48, p=3.77e-15) showed positive correlation with the neutrophils, whereas TMM40 (r=-0.58,p=7.67e-23), CSNK2A2 (r=-0.43, p=2.33e-12), PGAM5 (r=-0.15, p=0.02), CSNK2B (r=-0.33, p=2.05e-07) and ATG12 (r=-0.07, p=0.31) were negatively correlated with neutrophils (**Figures 5B–I**). Based on the above results, we further explored the relationship between 28 inflammatory factors and 7 key mitophagy genes. As shown in **Figure 6**, MFN1 was negatively correlated with IL-10, CD4 and IFNB1 (*p*<0.001), TOMM40 was positively correlated with PDGFA, IL10 and CD4 (*p*<0.001), CSNK2A2 was negatively correlated with IL10, CD4 and FGFB3, and positively with MAP1LC3B (*p*<0.001), CSNK2A was negatively and positively correlated with IL-15 (*p*<0.001) and CD4 (*p*<0.001), PGAM5 was positively correlated with PDGFA (*p*<0.01), CSNK2B was positively correlated with PDGFA, HLA-DRB4, CD4 and HLA-DRB3 (*p*<0.001), and negatively with IFNG (*p*<0.001), and ATG12 was positively correlated with IFNG (*p*<0.001), and negatively correlated IL-10, CD4 and HLA-DRB3 (*p*<0.001).

### 3.4 Results of differential mitophagy genotypes

**Figure 7:**
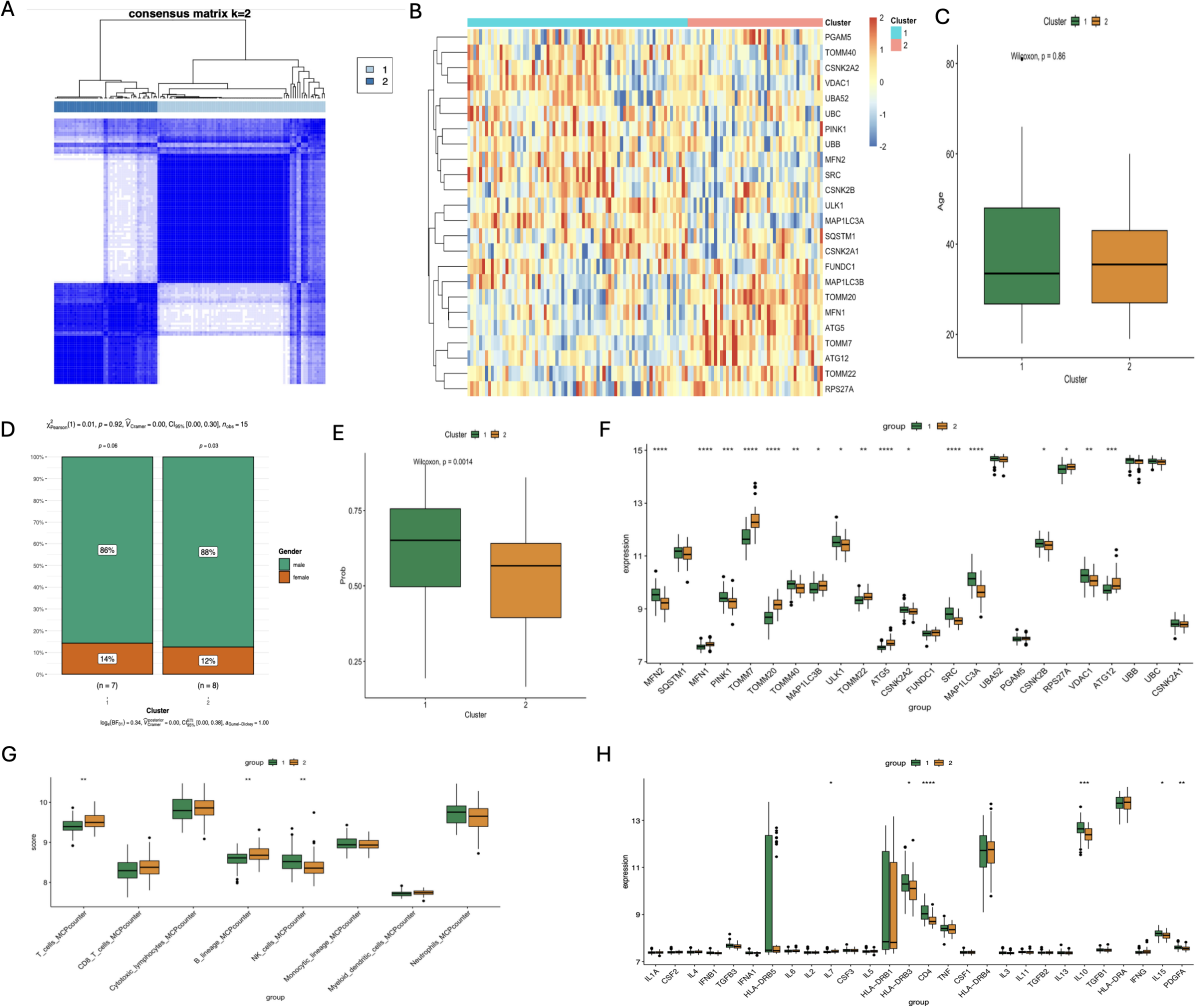
Analysis of different subgroups of SCZ patients based on Characteristics of mitophagy gene Expression and immunologic profile. **(A)** Correlation heatmap of 24 significantly different mitophagy genes in the two subtypes; **(B)** Age correlation analysis of the two subtypes; **(C)** Sex-related analysis of the two subtypes; **(D)** Correlation analysis of the clinical diagnosis prediction model constructed by core genes of the two subtypes; **(E)** Bar chart of differential expression analysis of 24 significantly different mitophagy genes in the two subtypes; **(F)** Differential analysis of immune cell infiltration scores between the two subtypess; **(G)** Differential expression analysis of inflammatory factors in the two subtypes. (*p < 0.05; **p < 0.01; ***p < 0.001; ****p < 0.0001).

Through clustering analysis, the schizophrenia patients were divided into two distinct mitophagy clusters - C1 and C2 - which were relatively stable as per the cumulative distribution function (CDF) curves and delta area (**Figure 7A**). The C1 and C2 populations differed significantly in terms of the expression of mitophagy genes (**Figure 7B**), but showed no significant differences in clinical features like age or sex (**Figures 7C, D**). Interestingly, the C1 cluster showed higher predictive accuracy compared to C2, indicating that individuals classified as C1 are more likely to develop schizophrenia (p=0.0014; **Figure 7E**). TOMM40 and CSNK2B were significantly activated in C1, whereas TOMM20, MFN1, and ATG5 were activated in C2 (**Figure 7F**). In addition, while T cells and B cells were more abundant in C2, the NK cells had higher abundance in C1 (**Figure 7G**). The pro-inflammatory factor IL-7 was increased in C2, and HLA-DRB3, CD4, IL-10, IL-15, and PDGFA were increased in C1 (**Figure 7H**). Our results indicate a possible association between mitophagy and inflammatory responses in schizophrenia, which merits further investigation.

### 3.5 Multiple cluster analyses

**Figure 8:**
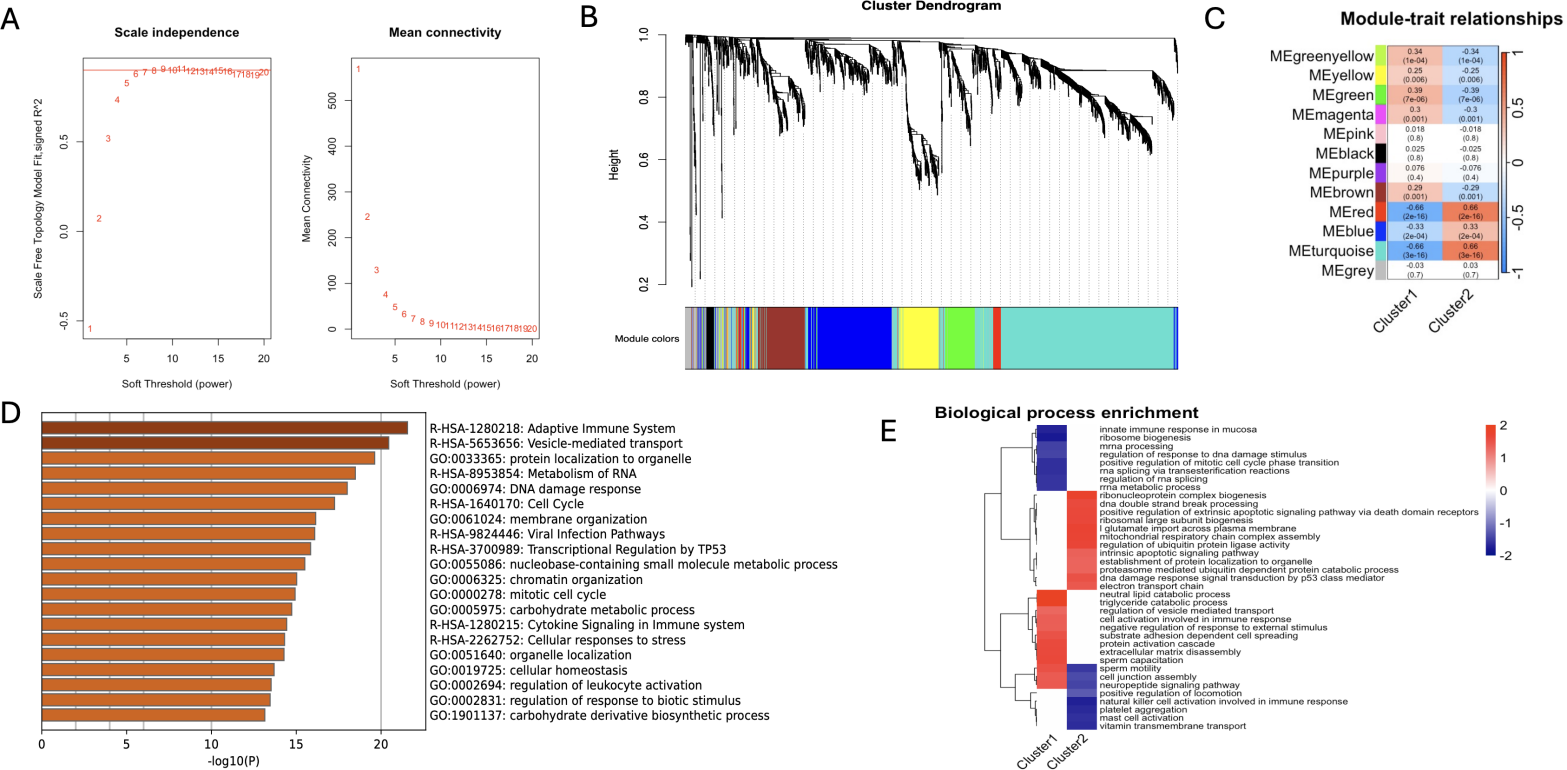
Results of WGCNA and fGSEA analysis. **(A)** After consistency scoring, the SCZ samples were divided into two stable types: Cluster1 and Cluster2; **(B)** Scale independence (left panel) and mean connectivity (right panel) of WGCNA analysis; **(C)** Feature association maps between 12 modules and two types of SCZ samples; **(D)** GO analysis bar chart of key genes in e.ed module; **(E)** Cluster plots of Biological process enrichment analysis of two types of SCZ samples.

Weighted gene co-expression network analysis (WGCNA) was performed on the combined data set to screen the co-expressed genes related to schizophrenia. The scale independence and mean connectivity for WGCNA are shown in **Figure 8A**. We constructed co-expression networks based on optimal soft thresholds and plotted gene clustering trees (**Figure 8B**). An association analysis was performed using heat maps for each sample and the identified modules. As shown in **Figure 8C**, the MEred module was significantly correlated to C1 (r=-0.66, p=5e-16) and C2 (r=0.66, p=5e-16). GO analysis of genes in the red module revealed significant enrichment of adaptive immune system, cytokine signaling transduction in the immune system, regulation of leukocyte activation and other pathways related to immune response (**Figure 8D**). We also compared the pathways between C1 and C2 through fGSEA, and found that C1 was mainly enriched in mucosal innate immune response, cell activation involved in immune response and other immune response pathways, while C2 was enriched in metabolic signaling pathways such as mitochondrial respiratory chain complex assembly and neuropeptide signaling pathway (**Figure 8E**). These results highlight the potential impact of neuroimmunity regulated by mitophagy genes on clinical intervention and drug development for schizophrenia.

### 3.6 Comprehensive single-cell analysis

**Figure 9:**
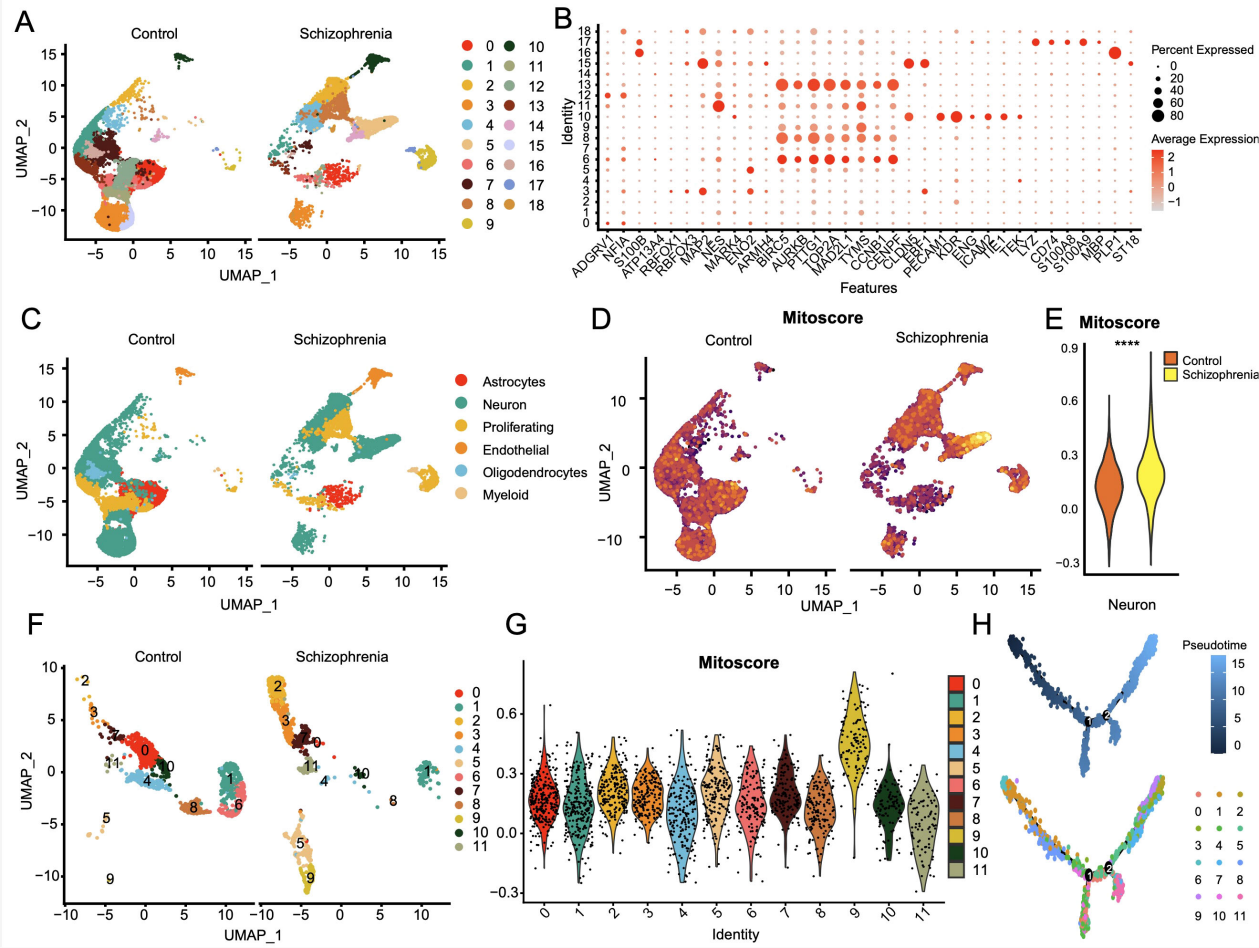
Single-cell profiles of SCZ versus CT donor-derived 3D brain organoids. **(A)** The single-cell clustering results of CT and SCZ identified UMAP maps of 19 cell clusters. **(B)** Bubble plot of expression profiles of significantly tagged genes in each subcluster. **(C)** Single cell principal component analysis mainly focused on six components: astrocytes, neurons, proliferative cells, endothelial cells, oligodendrocytes and myeloid cells; **(D)** Two UMAP plots of neuronal mitophagy scores; **(E)** Significant differences in the mitophagy scores of neuronal subsets between SCZ and CT groups, *p*< 0.0001; **(F)** 12 neuronal cell subsets obtained by single cell dimension reduction annotation of neurons; **(G)** Violin plot of mitophagy scores for 12 neural cell subsets; **(H)** Reverse chronological analysis of the neuronal subpopulation with a mitophagy score, the ninth group with a high mitophagy score was mainly at the end of the developmental trajectory.

**Figure 10:**
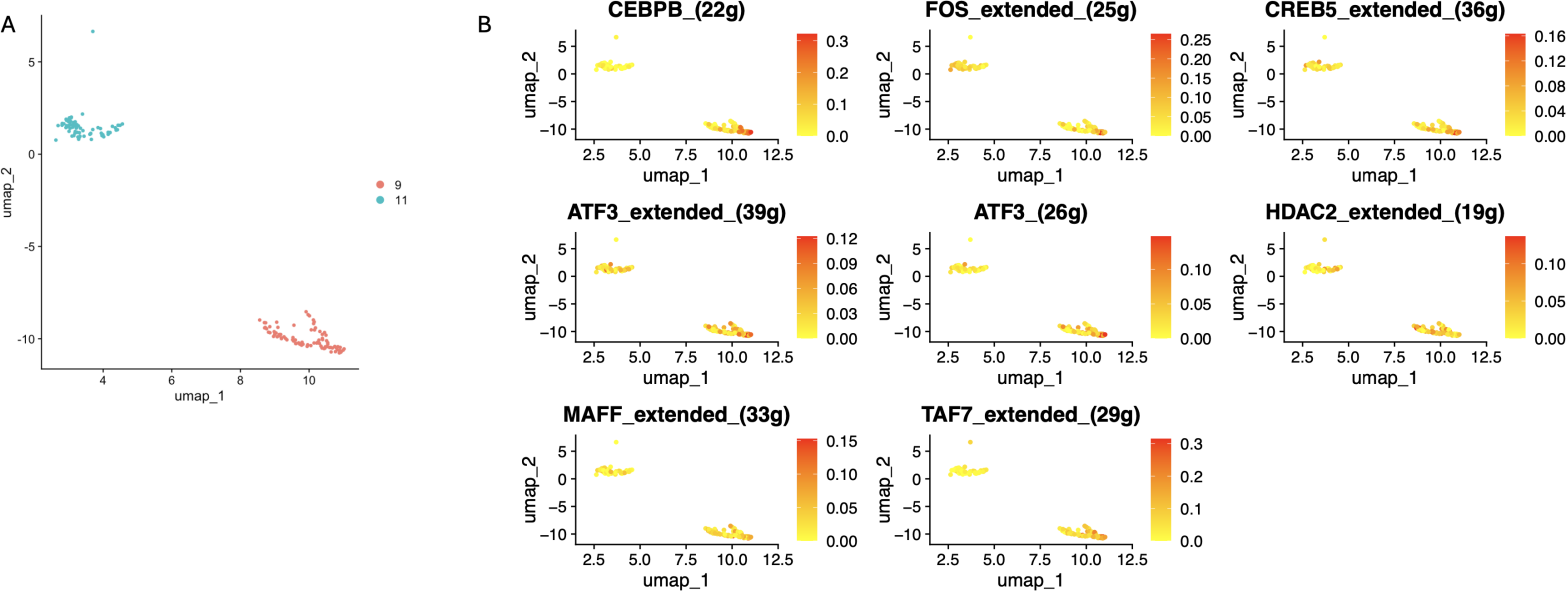
Differential distributionof subcellular clusters of specific transcription factors. **(A)** The 9th cluster with the high Mitoscore and the 11th cluster with the low Mitoscore. **(B)** Differences in distribution of seven specific transcription factors in subcellular clusters 9 and 11.

We identified 19 sub-clusters based on the scRNA-seq data (**Figure 9A**), and found that hypervariable genes were most prominent in subclusters 6, 8, and 13 (**Figure 9B**). Single-cell PCA identified six major cell populations, namely astrocytes, neurons, proliferating cells, endothelial cells, oligodendrocytes, and myeloid cells (**Figure 9C**). The neurons were the predominant population and therefore selected for subsequent analysis. The neurons were classified using Mitoscore (**Figure 9D**), and as shown in **Figure 9E**, the Mito_Neuron score was significantly higher in the schizophrenia group compared to the control group (p<0.0001). This suggested a potential role of neurons in the pathogenesis of schizophrenia. We further identified 12 sub-clusters of neurons using single cell dimensionality reduction (**Figure 9F**), of which cluster 9 had the highest mitophagy score (**Figure 9G**), whereas cluster 11 had the lowest score (**Figure 10A**). Pseudotime analysis further showed that higher Mitoscores mainly occurred at the end of the developmental trajectory of cluster 9, suggesting that these cells were unique to schizophrenia (**Figure 9H**). We next compared the expression levels of various transcription factors, including CEBPB, FOS, CREB5, ATF3, HDAC2, MAFF and TAF7, between clusters 9 (Mitohigh_Neuron) and 10 (Mitolow_Neuron). As shown in **Figure 10B**, CEBPB and TAF7 were highly expressed in Mitohigh_Neuron, while FOS and CREB5 were expressed at higher levels in Mitolow_Neuron. Given the role of these transcription factors in the immune response, we surmise that mitophagy may contribute to disease pathogenesis by regulating the immune response.

**Figure 11:**
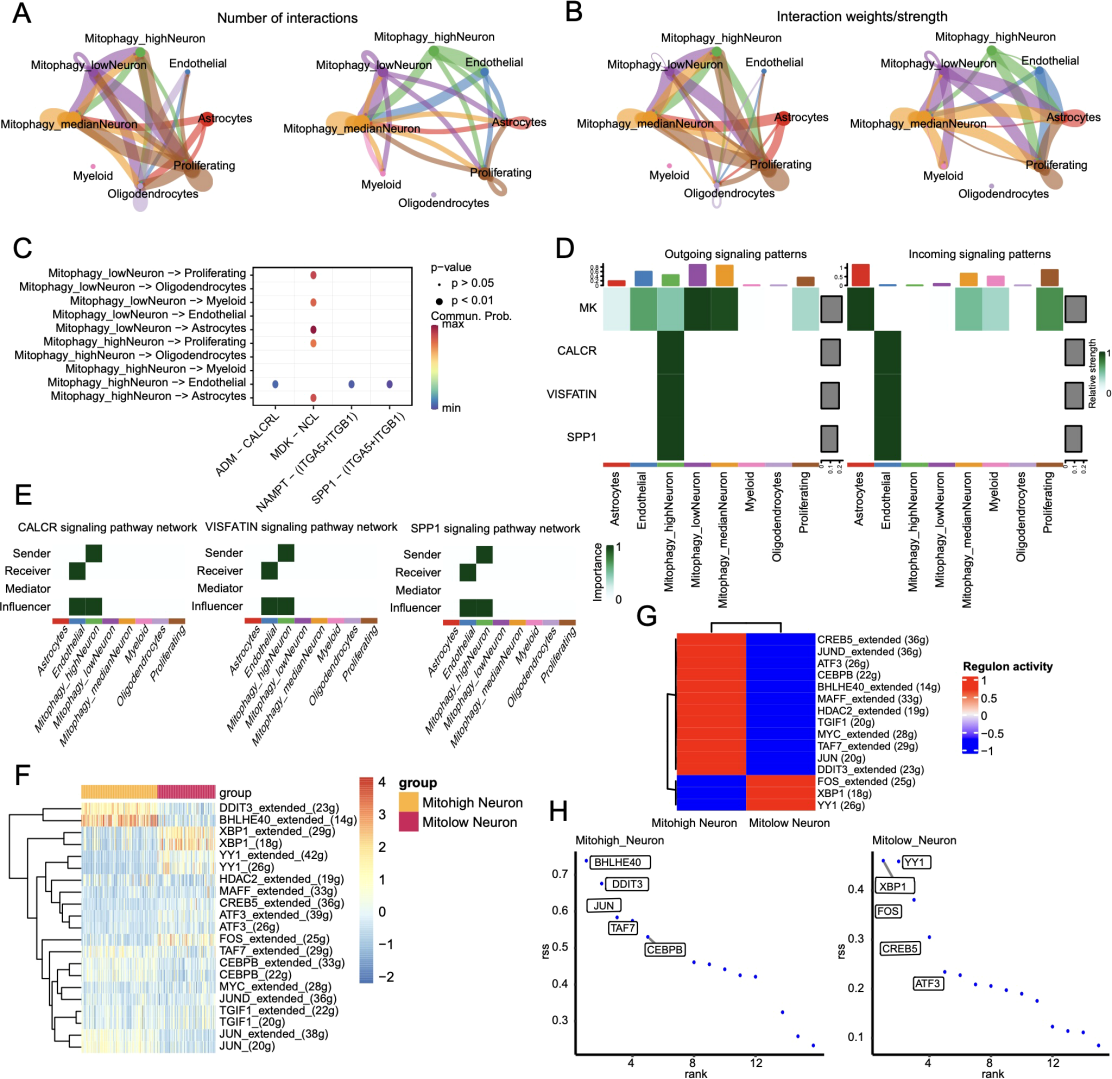
Cell communication results. **(A)**Statistical analysis of the number of interactions among cell populations. Cells expressing ligands are indicated by outgoing arrows, while cells expressing receptors are indicated by arrows pointing towards them; **(B)** Probability/strength values of interactions (strength is the sum of probability values); **(C)** Visualization of interactions between multiple ligand-receptor mediated cell relationships in a bubble chart; **(D)** Cell signaling flow patterns, with cell types on the horizontal axis and pathways on the vertical axis. **(E)** Heat map of interactions between cells mediated by ligand-receptor signaling pathways CALCR, VISFATIN, and SPP1; **(F)** Heat map of significant differential transcription factor Area Under the Curve (AUC) values in cell subgroups; **(G)** Average regulatory activity of significant differential transcription factors in two cell subclusters; **(H)** Scatter plot of specific transcription factor scoring indices for two cell subgroups.

Cell communication analysis was performed using the CellChat package. The number and strength of cell interactions are shown in **Figures 11A, B**, and the bubble plots of ligand-receptor interactions for the 10 mitophagy subclusters are shown in **Figure 11C**. The Mitohigh_Neuron-endothelial and Mitolow_Neuron-endothelial interactions showed significant differences in the ADMM-CALCRLHE, NAMPT-(ITGA5+ITGB1), and SPP1-(ITGA5+ITGB1) signaling pathways. In addition, Mitohigh_Neuron and endothelial cells showed enhanced SPP1 and NAMPT signals, which were mainly contributed by the CALCR, VISFATIN, and SPP1 signaling pathways (**Figure 11D**). Consistent with this, Mitohigh_Neuron was associated with all three signaling pathways (**Figure 11E**).

**Figure 12:**
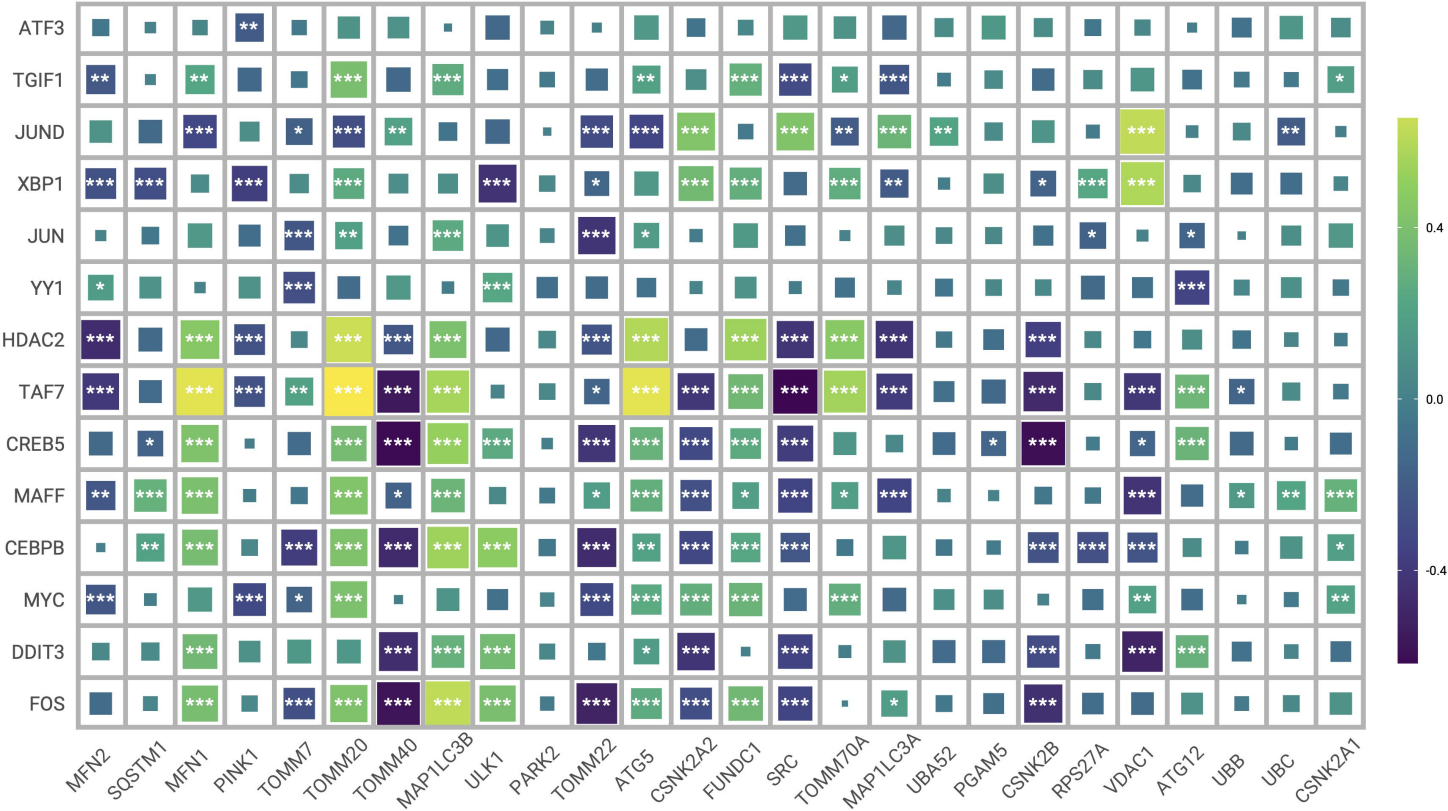
Heat map of correlation between transcription factors and mitophagy genes. (*P ≤ 0.05; **P ≤ 0.01; ***P ≤ 0.001).

The regulatory networks of the significantly different transcription factors between Mitohigh_Neuron and Mitolow_Neuron subsets were analyzed using SCENIC (**Figure 11F**). As shown in **Figure 11G**, BHLHE40 had the highest specificity in the Mitohigh_Neuron group, and YY1 had the highest specificity in the Mitolow_Neuron group. DDIT3_extended and BHLHE40_extended were significantly up-regulated in the Mitohigh_Neuron group, while XBP1_extended and FOS_extended were significantly upregulated in the Mitolow_Neuron group (**Figure 11H**). Furthermore, DDIT3, TAF7 and CEBPB were positively correlated with MFN1 and TOMM20 in the Mitohigh_Neuro cluster, and negatively correlated with CSNK2A2 and CSNK2B. In the Mitolow_Neuro cluster, XBP1 showed a significant positive correlation with CSNK2A2 and PINK1, and YY1 was negatively correlated with ATG12 (**Figure 12**). Taken together, mitophagy genes may affect schizophrenia progression by regulating specific transcription factors.

## 4 Discussion

Schizophrenia patients exhibit changes in brain structure, functional connectivity, nerve cells, and immune cells compared to healthy subjects, which may directly affect disease progression and symptoms (29–31). For instance, the cerebral neurons of schizophrenia patients often appear structurally aberrant, with abnormal size, dendritic and synaptic structures, etc., which may directly affect the transmission of signals within the neural network (32, 33). The resulting dysfunctional coordination between different brain regions can manifest as altered perception, emotion, cognition, etc (34). In addition, schizophrenia patients often experience changes in the volume of frontal lobe, temporal lobe, parietal lobe, and other brain regions, which may be associated with the symptoms and cognitive dysfunction of schizophrenia (35). Moreover, a dysregulated neuroimmune response in schizophrenia patients may trigger an inflammatory response that affects neuronal function and brain homeostasis (36, 37).

Mitochondria are particularly abundant in neurons due to their high energy requirements for maintaining normal electrophysiological activity and synaptic transmission (38, 39). Therefore, structural and functional abnormalities in the mitochondria may play an important role in the occurrence and development of schizophrenia by affecting the neurons. In fact, mitochondrial abnormalities, such as irregular morphology and distribution in neurons, have been observed in the brain tissues of schizophrenia patients (7). Mitochondrial dysfunction manifests as impaired respiratory chain, mitochondrial DNA damage, and depolarization of the mitochondrial membrane, which culminate in elevated intracellular oxidative stress (40, 41). Mitochondria-induced oxidative damage in the neurons affects their signaling and function. Mitophagy plays an important role in maintaining mitochondrial homeostasis and removing damaged mitochondria (42). Any dysregulation in the mitophagy process may lead to excessive accumulation of damaged mitochondria, resulting in neuronal apoptosis and abnormal brain function that may progress to schizophrenia (43). Consistent with the above hypothesis, we observed significant differences in the expression of mitophagy genes between the schizophrenia patients and healthy controls. Alternatively, differentially expressed mitophagy genes identified in both blood samples and brain organoids reveal systemic and localized alterations in schizophrenia. The expression patterns of these genes in blood samples may reflect broader systemic changes, while those in brain organoids provide insight into local disruptions within the central nervous system. Finally, we identified CSNK2B, TOMM40, MAP1LC3B, MFN1, CSNK2A2, PGAM5 and ATG12 as the diagnostic genes for schizophrenia. The nomogram based on these genes showed good diagnostic performance.

The expression of mitophagy genes was also correlated with most immune cells, including neutrophils, activated DCs, activated NK cells, resting NK cells, regulatory T cells, activated CD4 memory T cells and CD8 T cells. In particular, MFN1 and MAP1LC3B were positively correlated with the neutrophils, whereas TMM40, CSNK2A2, PGAM5 and CSNK2B showed a negative correlation. By consistency cluster analysis, schizophrenia patients were divided into two subgroups with different mitophagy gene expression and immunological characteristics. There were no significant differences in clinical manifestations between C1 and C2 subsets, but there were differences in the distribution of immune cells and the expression of inflammatory factors. Subgroup C1 showed higher prediction accuracy, suggesting that individuals in this subgroup are more likely to develop schizophrenia.

We then identified 12 sub-clusters of neurons using single cell dimensionality reduction. Furthermore, the proportion of type E endothelial cells was significantly elevated in schizophrenia patients, while the proportion of macrophages was significantly reduced. In addition, 15 hypervariable genes, including MUC5B, FDCSP, SCGB1A1, PRB4, ZG16B and BPIFA, were closely related to each cell cluster. The expression of seven characteristic mitophagy genes were also significantly different among the nine clusters. Taken together, schizophrenia is associated with considerable heterogeneity in transcriptomic and immunological profiles. Our findings suggest a potential role for mitophagy in disease progression, highlighting the need for further research to elucidate its impact.

CSNK2A2 and CSNK2B are protein kinases that regulate mitophagy through a ubiquitin-independent pathway. CSNK2A2 phosphorylates acidic proteins and is involved in the regulation of cell cycle, apoptosis, and circadian rhythm (44). CSNK2B catalyzes the phosphorylation of other proteins and participates in the regulation of cell growth, differentiation, apoptosis, and other biological processes (45). TOMM40 encodes the mitochondrial outer membrane protein TOM40, and various single nucleotide polymorphisms (SNPS) of TOMM40 are associated with mitochondrial dysfunction and neuropsychiatric disorders (46). MFN1 is located in the inner and outer mitochondrial membranes and maintains organelle morphology. Downregulation of MFN1 can sensitize neurons to apoptosis and impair cerebral cortex development (47). MAP1LC3B is a key component of autophagy and regulates the quantity and quality of mitochondria to meet cellular energy demands and prevent excessive ROS production (48). PGAM5 is involved in inflammatory responses, mitosis, apoptosis, lipid metabolism and other processes, and plays an important role in neurodegenerative diseases and ischemia-reperfusion injury (49). PGAM5 induces mitophagy in response to hypoxia by dephosphorylating FUNDC1 and is also involved in PINK1/Parkin mediated mitophagy (50). ATG12 is a key gene in the autophagy pathway, and its encoded ATG12 protein forms a complex with ATG5 and ATG16L1 proteins to regulate the early formation and extension of autophagosome (51). Overexpression of ATG12 inhibits neuronal axonal development *in vivo* (52).

In summay, Our study explored the disruption of mitophagy in schizophrenia based on comprehensive single-cell and batch RNA sequencing data combined with machine learning analysis. The subsets of neurons that we identified on the basis of Mitoscore (Mitohigh_Neuron, Mitomedian_Neuron, and Mitolow_Neuron) showed distinct patterns of mitophagy gene expression. In particular, the novel interaction between Mitohigh_Neuron subsets and endothelial cells, established through SPP1 signaling, may be a potential molecular mechanism pathway in schizophrenia.(2(Our analysis also highlights the role of mitophagy in immune cells. The expression patterns of mitophagy genes in these cells suggest a potential impact on the immune system’s functioning, which could influence neuroinflammation and immune responses associated with schizophrenia. Dysregulated mitophagy in immune cells may lead to altered immune cell activation and infiltration, exacerbating the inflammatory environment in the brain and contributing to disease progression.(3(Both neuronal and immune cell disruptions in mitophagy likely contribute to schizophrenia pathogenesis. The interplay between these cell types could create a feedback loop where neuronal dysfunction exacerbates immune dysregulation and vice versa.

However, there are several limitations of our study that need to be considered. Firstly, the study’s reliance on publicly available datasets may introduce biases related to sample selection and data quality. Secondly, the single cell and RNA sequencing data used in this study are from different data sets, and technical differences and biological heterogeneity between these data sets may affect the analysis results. Thirdly, our single-cell analysis of schizophrenia patients and controls was based on 3D brain organoids, and different sample-derived cells could potentially influence the results.In addition, while our analysis included blood and brain organoids, the expression of mitophagy genes in other cell types, such as glial cells, endothelial cells, and peripheral tissues, remains unexplored. These cells could potentially contribute to the mitophagy signature observed in schizophrenia, and future studies should investigate their roles. Finally, Finally, experimental validation of our findings is necessary to confirm the functional relevance of the identified mitophagy genes and their interactions with immune cells in the context of schizophrenia.

## 5 Conclusion

In conclusion, our work has identified seven key mitophage genes critically involved in SCZ progression.These genes provides new insights into the pathological mechanisms and potential targets for SCZ.

## Data Availability

The raw data supporting the findings of this article were retrieved from the NCBI Gene Expression Omnibus (GEO) database, accession numbers GSE38484, GSE38481 and GSE184878.

## References

[1] Jauhar S Johnstone M McKenna PJ. Schizophrenia. Lancet. (2022) 399:473–86. doi: 10.1016/S0140-6736(21)01730-X 35093231

[2] Wainschtein P Jain D Zheng Z TOPMed Anthropometry Working Group NHLBI Trans-Omics for Precision Medicine (TOPMed) Consortium Cupples LA. Assessing the contribution of rare variants to complex trait heritability from whole-genome sequence data. Nat Genet. (2022) 54:263–73. doi: 10.1038/s41588-021-00997-7 PMC9119698 35256806

[3] Momozawa Y Mizukami K. Unique roles of rare variants in the genetics of complex diseases in humans. J Hum Genet. (2021) 66:11–23. doi: 10.1038/s10038-020-00845-2 32948841 PMC7728599

[4] Owen MJ Legge SE Rees Walters JTR O'Donovan MC. Genomic findings in schizophrenia and their implications. Mol Psychiatry. (2023) 28:3638–47. doi: 10.1038/s41380-023-02293-8 PMC10730422 37853064

[5] Roberts RC. Mitochondrial dysfunction in schizophrenia: With a focus on postmortem studies. Mitochondrion. (2021) 56:91–101. doi: 10.1016/j.mito.2020.11.009 33221354 PMC7810242

[6] Ni P Chung S. Mitochondrial dysfunction in schizophrenia. Bioessays. (2020) 42:e1900202. doi: 10.1002/bies.201900202 32338416

[7] Chen W Zhao H Li Y. Mitochondrial dynamics in health and disease: mechanisms and potential targets. Sig Transduct Target Ther. (2023) 8:333. doi: 10.1038/s41392-023-01547-9 PMC10480456 37669960

[8] Brand MD Orr AL Perevoshchikova IV Quinlan CL. The role of mitochondrial function and cellular bioenergetics in ageing and disease. Br J Dermatol. (2013) 169 Suppl 2:1–8. doi: 10.1111/bjd.12208 PMC4321783 23786614

[9] Andreazza AC Nierenberg AA. Mitochondrial dysfunction: at the core of psychiatric disorders? Biol Psychiatry. (2018) 83:718–9. doi: 10.1016/j.biopsych.2018.03.004 29628041

[10] Manji H Kato T Di Prospero NA Ness S Beal MF Krams M. Impaired mitochondrial function in psychiatric disorders. Nat Rev Neurosci. (2012) 13:293–307. doi: 10.1038/nrn3229 22510887

[11] Gonçalves VF Andreazza AC Kennedy JL. Mitochondrial dysfunction in schizophrenia: an evolutionary perspective. Hum Genet. (2015) 134:13–21. doi: 10.1007/s00439-014-1491-8 25312050

[12] Rose S Niyazov DM Rossignol DA Goldenthal M Kahler SG Frye RE. Clinical and molecular characteristics of mitochondrial dysfunction in autism spectrum disorder. Mol Diagn Ther. (2018) 22:571–93. doi: 10.1007/s40291-018-0352-x PMC6132446 30039193

[13] Shivakumar V Rajasekaran A Subbanna M Kalmady SV Venugopal D Agrawal R. Leukocyte mitochondrial DNA copy number in schizophrenia. Asian J Psychiatr. (2020) 53:102193. doi: 10.1016/j.ajp.2020.102193 32585632

[14] Ni P Ma Y Chung S. Mitochondrial dysfunction in psychiatric disorders. Schizophr Res. (2022), S0920–9964(22)00333-4. doi: 10.1016/j.schres.2022.08.027 PMC12180538 36175250

[15] Sebastian R Song Y Pak C. Probing the molecular and cellular pathological mechanisms of schizophrenia using human induced pluripotent stem cell models. Schizophr Res. (2022), S0920–9964(22)00263-8. doi: 10.1016/j.schres.2022.06.028 PMC9832179 35835709

[16] Mootha V Lindgren C Eriksson KF Subramanian A Sihag S Lehar J. PGC-1α-responsive genes involved in oxidative phosphorylation are coordinately downregulated in human diabetes. Nat Genet. (2003) 34:267–73. doi: 10.1038/ng1180 12808457

[17] Ritchie ME Phipson B Wu D Hu Y Law CW Shi W. limma powers differential expression analyses for RNA-sequencing and microarray studies. Nucleic Acids Res. (2015) 43:e47. doi: 10.1093/nar/gkv007 25605792 PMC4402510

[18] Newman AM Liu CL Green MR Gentles AJ Feng W Xu Y. Robust enumeration of cell subsets from tissue expression profiles. Nat Methods. (2015) 12:453–7. doi: 10.1038/nmeth.3337 PMC4739640 25822800

[19] Zeng D Ye Z Shen R Yu G Wu J Xiong Y. IOBR: multi-omics immuno-oncology biological research to decode tumor microenvironment and signatures. Front Immunol. (2021) 12:687975. doi: 10.3389/fimmu.2021.687975 34276676 PMC8283787

[20] Wickham H. g gplot2: Elegant Graphics for Data Analysis [Internet]. 2nd ed. Springer. (2016). doi: 10.1007/978-3-319-24277-4

[21] Simonsen AT Hansen MC Kjeldsen E Møller PL Hindkjær JJ Hokland P. Systematic evaluation of signal-to-noise ratio in variant detection from single cell genome multiple displacement amplification and exome sequencing. BMC Genomics. (2018) 19:681. doi: 10.1186/s12864-018-5063-5 30223769 PMC6142419

[22] Wilkerson MD Hayes DN. ConsensusClusterPlus: a class discovery tool with confidence assessments and item tracking. Bioinformatics. (2010) 26:1572–3. doi: 10.1093/bioinformatics/btq170 PMC2881355 20427518

[23] Langfelder P Horvath S. WGCNA: an R package for weighted correlation network analysis. BMC Bioinf. (2008) 12:559. doi: 10.1186/1471-2105-9-559 PMC2631488 19114008

[24] Huang Q Liu Y Du Y Garmire LX. Evaluation of cell type annotation R packages on single-cell RNA-seq data. Genomics Proteomics Bioinf. (2021) 19:267–81. doi: 10.1016/j.gpb.2020.07.004 PMC8602772 33359678

[25] Liberzon A Birger C Thorvaldsdóttir H Ghandi M Mesirov JP Tamayo P. The Molecular Signatures Database (MSigDB) hallmark gene set collection. Cell Syst. (2015) 1:417–25. doi: 10.1016/j.cels.2015.12.004 PMC4707969 26771021

[26] Qiu X Hill A Packer J Lin D Ma YA Trapnell C. Single-cell mRNA quantification and differential analysis with Census. Nat Methods. (2017) 14:309–15. doi: 10.1038/nmeth.4150 PMC5330805 28114287

[27] Leshchevnikov V Shmatko A Dann E Aivazidis A King HW Li T. Cell2location maps fine-grained cell types in spatial transcriptomics. Nat Biotechnol Nat Biotechnol. (2022) 40:661–71. doi: 10.1038/s41587-021-01139-4 35027729

[28] Li H Sun Y Hong H Huang X Tao H Huang QY. Inferring transcription factor regulatory networks from single-cell ATAC-seq data based on graph neural networks. Nat Mach Intell. (2022) 4:389–400. doi: 10.1038/s42256-022-00469-5

[29] Wang YM Cai XL Zhang RT Zhang YJ Zhou HY Wang Y. Altered brain structural and functional connectivity in schizotypy. Psychol Med. (2022) 52:834–43. doi: 10.1017/S0033291720002445 32677599

[30] Zhang J Yang Y Liu T Shi Z Pei G Wang L. Functional connectivity in people at clinical and familial high risk for schizophrenia. Psychiatry Res. (2023) 328:115464. doi: 10.1016/j.psychres.2023.115464 37690192

[31] Lv Y Wen L Hu WJ Deng C Ren HW Bao YN. Schizophrenia in the genetic era: a review from development history, clinical features and genomic research approaches to insights of susceptibility genes. Metab Brain Dis. (2024) 39:147–71. doi: 10.1007/s11011-023-01271-x 37542622

[32] Kraguljac NV Lahti AC. Neuroimaging as a window into the pathophysiological mechanisms of schizophrenia. Front Psychiatry. (2021) 12:613764. doi: 10.3389/fpsyt.2021.613764 33776813 PMC7991588

[33] Kong L Herold CJ Cheung EFC Chan RCK Schröder J. Neurological soft signs and brain network abnormalities in schizophrenia. Schizophr Bull. (2020) 46:562–71. doi: 10.1093/schbul/sbz118 PMC7147582 31773162

[34] Hollunder B Ostrem JL Sahin IA Rajamani N Oxenford S Butenko K. Mapping dysfunctional circuits in the frontal cortex using deep brain stimulation. Nat Neurosci. (2024) 27:573–86. doi: 10.1038/s41593-024-01570-1 PMC10917675 38388734

[35] Premkumar P Kumari V Corr PJ Sharma T. Frontal lobe volumes in schizophrenia: effects of stage and duration of illness. J Psychiatr Res. (2006) 40:627–37. doi: 10.1016/j.jpsychires.2006.05.009 16901506

[36] Comer AL Carrier M Tremblay MÈ Cruz-Martín A. The inflamed brain in schizophrenia: the convergence of genetic and environmental risk factors that lead to uncontrolled neuroinflammation. Front Cell Neurosci. (2020) 14:274. doi: 10.3389/fncel.2020.00274 33061891 PMC7518314

[37] Murphy CE Walker AK Weickert CS. Neuroinflammation in schizophrenia: the role of nuclear factor kappa B. Transl Psychiatry. (2021) 11:528. doi: 10.1038/s41398-021-01607-0 34650030 PMC8516884

[38] Trigo D Vitória JoséJoão da Cruz e Silva OB. Novel therapeutic strategies targeting mitochondria as a gateway in neurodegeneration. Neural Regeneration Res. (2023) 18:991–5. doi: 10.4103/1673-5374.355750.4 PMC9827793 36254979

[39] Pei Y Chen S Zhou F Xie T Cao H. Construction and evaluation of Alzheimer’s disease diagnostic prediction model based on genes involved in mitophagy. Front Aging Neurosci. (2023) 15:1146660. doi: 10.3389/fnagi.2023.1146660 37032823 PMC10077494

[40] Zhou WC Qu J Xie SY Sun Y Yao HW. Mitochondrial dysfunction in chronic respiratory diseases: implications for the pathogenesis and potential therapeutics. Oxid Med Cell Longev. (2021) 2021:5188306. doi: 10.1155/2021/5188306 34354793 PMC8331273

[41] Danieli MG Antonelli E Piga MA Cozzi MF Allegra A Gangemi S. Oxidative stress, mitochondrial dysfunction, and respiratory chain enzyme defects in inflammatory myopathies. Autoimmun Rev. (2023) 22:103308. doi: 10.1016/j.autrev.2023.103308 36822387

[42] Ma K Chen G Li W Kepp O Zhu Y Chen Q. Mitophagy, mitochondrial homeostasis, and cell fate. Front Cell Dev Biol. (2020) 8:467. doi: 10.3389/fcell.2020.00467 32671064 PMC7326955

[43] Clemente-Suárez VJ Redondo-Flórez L Beltrán-Velasco AI Ramos-Campo DJ Belinchón-deMiguel P Martinez-Guardado I. Mitochondria and brain disease: A comprehensive review of pathological mechanisms and therapeutic opportunities. Biomedicines. (2023) 11:2488. doi: 10.3390/biomedicines11092488 37760929 PMC10526226

[44] Chen WH Lin YX Lin L Zhang BQ Xu SX Wang W. Identification of potential candidate proteins for reprogramming spinal cord-derived astrocytes into neurons: a proteomic analysis. Neural Regener Res. (2021) 16:2257–63. doi: 10.4103/1673-5374.310697 PMC8354129 33818510

[45] Yang CP Li X Wu Y Shen Q Zeng Y Xiong Q. Comprehensive integrative analyses identify GLT8D1 and CSNK2B as schizophrenia risk genes. Nat Commun. (2018) 9:838. doi: 10.1038/s41467-018-03247-3 29483533 PMC5826945

[46] Chen S Sarasua SM Davis NJ DeLuca JM Boccuto L Thielke SM. TOMM40 genetic variants associated with healthy aging and longevity: a systematic review. BMC Geriatr. (2022) 22:667. doi: 10.1186/s12877-022-03337-4 35964003 PMC9375314

[47] Choudhury M Fu T Amoah K Jun HI Chan TW Park S. Widespread RNA hypoediting in schizophrenia and its relevance to mitochondrial function. Sci Adv. (2023) 9:eade9997. doi: 10.1126/sciadv.ade9997 37027465 PMC10081846

[48] Bonam SR Bayry J Tschan MP Muller S. Progress and challenges in the use of MAP1LC3 as a legitimate marker for measuring dynamic autophagy in vivo. Cells. (2020) 9:1321. doi: 10.3390/cells9051321 32466347 PMC7291013

[49] Liang MZ Lu TH Chen L. Timely expression of PGAM5 and its cleavage control mitochondrial homeostasis during neurite re-growth after traumatic brain injury. Cell Biosci. (2023) 13:96. doi: 10.1186/s13578-023-01052-0 37221611 PMC10207772

[50] Cheng M Lin N Dong D Ma J Su J Sun L. PGAM5: A crucial role in mitochondrial dynamics and programmed cell death. Eur J Cell Biol. (2021) 100:151144. doi: 10.1016/j.ejcb.2020.151144 33370650

[51] Lystad AH Carlsson SR Simonsen A. Toward the function of mammalian ATG12-ATG5-ATG16L1 complex in autophagy and related processes. Autophagy. (2019) 15:1485–6. doi: 10.1080/15548627.2019.1618100 PMC6613903 31122169

[52] Yang K Yu B Cheng C Cheng T Yuan B Li K. Mir505-3p regulates axonal development via inhibiting the autophagy pathway by targeting Atg12. Autophagy. (2017) 13:1679–96. doi: 10.1080/15548627.2017.1353841 PMC5640177 28820282

